# Abnormal Galectin Signaling in the Pathomechanisms of Placental Dysfunction in Gestational Diabetes Mellitus

**DOI:** 10.3390/ijms27052223

**Published:** 2026-02-26

**Authors:** Dariusz Szukiewicz

**Affiliations:** Department of Biophysics, Physiology & Pathophysiology, Faculty of Health Sciences, Medical University of Warsaw, 02-004 Warsaw, Poland; dariusz.szukiewicz@wum.edu.pl

**Keywords:** galectins, glycan binding proteins, placental galectins, galectin expression, gestational diabetes, placenta, placental dysfunction, angiogenesis, diabetic placenta

## Abstract

Recognition and binding to β-galactose-containing carbohydrates and lipids are crucial for several fundamental biological processes that are mediated primarily by a family of proteins known as galectins (S-type lectins). Galectins in the human placenta regulate critical processes such as maternal–fetal immune tolerance, trophoblast invasion, vascular remodeling and angiogenesis, ensuring proper fetal development and preventing pregnancy complications such as preeclampsia and miscarriage. Gestational diabetes mellitus (GDM) is a widespread complication of pregnancy, affecting approximately 1 in 7 pregnancies, and its incidence is increasing globally, indicating a particularly strong association with the obesity pandemic. Profiles of placental expression and distribution of individual galectins significantly change during the course of GDM. This is accompanied by placental dysfunction, which is especially severe with poor glycemic control. The aim of this review is to present the current state of knowledge on the involvement of abnormal galectin signaling in the pathomechanisms of GDM-associated placental dysfunction. Further research is needed to determine whether changes in placental galectins occur secondary to metabolic abnormalities in GDM or are involved as a primary cause. Galectins present in placental tissue and serum should be validated as potential biomarkers of GDM.

## 1. Introduction

The name galectin encompasses a class of proteins, identified in organisms from invertebrates to humans, that exhibit specific affinity for β-galactose-containing carbohydrates, such as N-acetyllactosamine (LacNac), a substrate for galactosidases. Galectin occurs in two primary structural forms, distinguished by the linkage between galactose and N-acetylglucosamine (GlcNAc): Galβ1-3GlcNAc (type 1) and Galβ1-4GlcNAc (type 2) [1,2]. Both forms of GlcNAc constitute fundamental building blocks in glycobiology, which can be bound to proteins by either N-linked or O-linked glycosylation and create biological scaffolds for many complex glycoproteins and glycolipids, which are crucial for poly-N-acetyllactosamine chains [3,4]. Galectins form lattices to cluster receptors, influencing signal strength, or bind directly to proteins such as B-cell lymphoma 2 (Bcl-2) protein to regulate programmed cell death (apoptosis) [5].

In addition to the control of apoptosis, the versatile carbohydrate-binding capacity of galectins allows them to participate in signaling related to numerous biological processes, both physiological and pathological, including the immune response with T-cell activation, inflammation, cell adhesion, migration and differentiation [5,6,7,8,9,10]. The action of galectins can be attributed to the function of “signalosomes”, which fine-tune cellular communication in health and disease [11]. The influence of galectins on basic cellular functions indicates that they play important roles in the pathomechanisms of cancer (tumor growth, metastasis), infections, productive inflammation with fibrosis, and degenerative changes in heart muscle (cardiomyopathies) [10,12,13,14,15,16,17,18].

Galectins are potent immunomodulators that regulate maternal immune responses during pregnancy and prevent rejection of the fetus as a semiallogeneic graft [19,20,21,22]. Variable expression and distribution of different galectins in placental tissue correlate with the establishment and regulation of maternal–fetal immune tolerance, the differentiation pathways of trophoblasts, the promotion of angiogenesis, and placental development [23,24,25,26,27].

Gestational diabetes mellitus (GDM) is generally defined as glucose intolerance manifested by hyperglycemia that is first diagnosed or develops during pregnancy [28,29]. GDM is the most common medical complication during pregnancy, affecting up to 1 in 7 pregnancies globally; increased rates of GDM are driven by increasing obesity, sedentary lifestyles, older maternal age, and genetic predispositions, along with improved detection due to widespread screening and changing guidelines [30,31]. GDM, especially with poorly controlled glycemia, poses significant risks for both the mother and fetus, including preeclampsia, fetal macrosomia, cesarean section, and neonatal hypoglycemia in the short term and maternal type 2 diabetes and obesity, metabolic syndrome, and diabetes later in the child’s life in the long term [31,32,33].

In GDM, galectins are dysregulated both in maternal blood and in the placenta, with significantly decreased placental galectin(Gal)-13 levels and upregulated Gal-1, Gal-3, Gal-4, Gal-7, Gal-9, and Gal-12 levels; additionally, galectins are being increasingly recognized for their significant involvement in GDM-related alterations in placental function [34,35,36,37,38,39,40,41]. Moreover, an abnormal galectin profile can be considered a potential early biomarker for GDM, indicating a disturbed cellular response to stress and an increased risk of inflammation and immune regulation disorders within the uteroplacental unit [19].

The aim of this review is to present the current state of knowledge on the involvement of abnormal galectin signaling in the pathomechanisms of GDM-associated placental dysfunction.

### Literature Search Methodology

A comprehensive review of the current state of knowledge was performed regarding the involvement of abnormal galectin signaling pathways in the pathomechanisms of placental structure and function disorders and ultimately pregnancy complications accompanying GDM. While trying to maintain maximum criticism and objectivity, a synthesis of relevant research and generalization of the collected data was made. This approach established a coherent framework for presenting findings, supporting a qualitative analysis of the topic and facilitating the achievement of the predefined objectives.

The main aim of the literature analysis was to check whether there are any associations between GDM, changes in the expression and function of galectins and placenta-related processes such as trophoblast invasion, placentation and initiation of uterine contractions (labor). To this end, data on the physiological significance of galectins in pregnancy were initially collected, limiting it as far as possible to humans and primates. The information obtained was then compared with estimates of the incidence and types of complications encountered in diabetic pregnancy. This approach enabled the identification of pathways and factors involved in the basic cellular functions of galectins in health and GDM, the latter also in clinical references.

Highly cited, peer-reviewed publications in journals from Master Journal List (MJL) in the fields of anatomy, physiology/pathophysiology, endocrinology/diabetology, obstetrics/placentology, immunology and cell biology/glycobiology were selected. In the case of review articles, an important selection criterion was their topicality (date of publication). The electronic databases PubMed, Scopus, Web of Science (WoS) and Google Scholar were reviewed. Priority was given to studies providing observational or experimental evidence.

The following relevant leading keywords/phrases were used: “glycan binding proteins”, “galectins”, “galectin signaling”, “placental galectins”, “galectin expression”, “galectin and trophoblast function”, “galectins and angiogenesis”, “galectins and placentation”, “galectins and placental dysfunction”, “diabetic placenta”, “gestational diabetes mellitus”.

The synthesis of the collected data aimed to explain the (patho)mechanisms through which disturbed galectin signaling pathways present in the human placenta influence the course of pregnancy complicated by GDM. The findings were then summarized and generalized to highlight important issues in classifying galectin changes as primary or secondary in GDM and the possibility of using specific galectins as potential biomarkers of GDM.

## 2. Galectins

Galectins (formerly known as “S-type lectins”) are a large conserved family of soluble carbohydrate-binding proteins (lectins) that recognize β-galactoside sugars and glycolipids inside the cell (cytosol/nucleus), on the surface of the cell membrane, and outside the cell (extracellular matrix) [2,42]. Their presence has also been demonstrated in the blood [2,42]. As already indicated in the introduction, the two main structural forms of galectins (Galβ1-3GlcNAc and Galβ1-4GlcNAc), formed as a result of bonds with β-galactoside sugars, participate in almost every biological process, including immune activity (e.g., maternal–fetal immune tolerance), cell growth/death (apoptosis), cancer progression (metastasis, angiogenesis), cell adhesion, development, and even pathogen recognition, by creating scaffolds for structural glycoproteins and glycolipids [13,42,43,44]. Galectins, often independent of their sugar-binding role, orchestrate angiogenesis during decidualization and placentation and regulate trophoblast development, migration, and invasion [45,46]. Consequently, altered expression of galectins is associated with infertility and an abnormal course of pregnancy [46].

### 2.1. Structure and General Characteristics of the Galectins Family

Humans have 12 distinct galectin genes/proteins, out of 16 identified in the animal kingdom and 15 in mammals, including Gal-1 through Gal-4, Gal-7, Gal-8, Gal-9, Gal-10, Gal-12, Gal-13, Gal-14, and Gal-16 [47]. The gene names, gene locations, and tissue distributions of human galectins, considering the uteroplacental unit, are listed in Table 1.

On the basis of the domain arrangement, these galectins can be classified into three types: ❶ prototype galectins, possessing a single carbohydrate recognition domain (CRD), which include Gal-1, Gal-2, Gal-7, Gal-10, Gal-13, Gal-14, and Gal-16, most often occurring in the form of dimers; ❷ tandem repeat-type galectins, with two CRDs in tandem, represented by Gal-4, Gal-8, Gal-9, and Gal-12; and ❸ chimeric-type galectins, with the only representative being Gal-3, whose CRD is fused to another nonlectin domain [62,81,82,83].

Tandem repeat-type galectins constitute a heterodimer consisting of two distinct monophyletic groups referred to as F3 and F4 CRD types, distinguished on the basis of sequence analysis and intron/exon positions [84].

Unlike other galectins that typically remain as monomers or dimers in solution, galectin-3, the sole member of the chimeric galectin family, occurring primarily as a monomer in solution, is also characterized by a unique ability to form pentamers or higher-order oligomers (lattices) with multivalent ligands to perform its biological function as a dynamic scaffold on cell surfaces [85,86,87,88].

**Figure 1:**
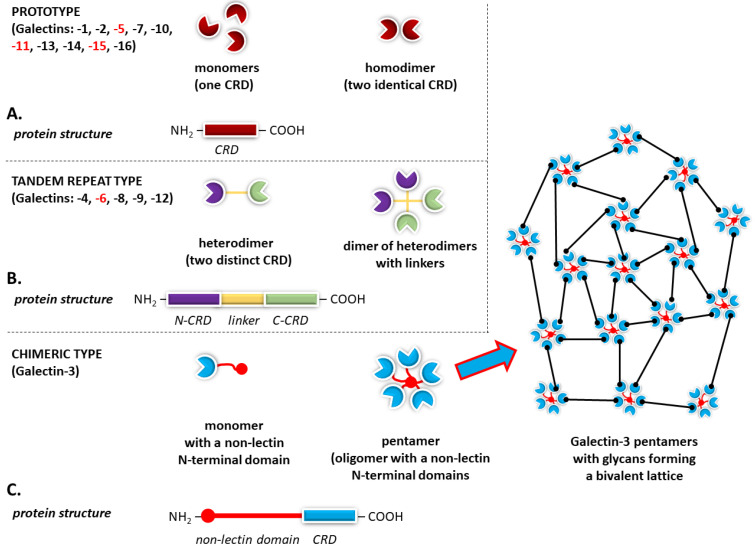
Classification of galectins based on protein structure. Of the 16 galectins known in the animal kingdom and 15 in mammals, 12 occur in humans (Gal-5, -6, -11 and -15, which are not found in humans, are marked in red). (**A**). Prototype galectins possess a single CRD and exist as monomers or non-covalently associated homodimers. (**B**). Two different but homologous CRDs, derived from a monophyletic group, in tandem repeat type galectins form a heterodimer with a functional linker or an oligomeric form (dimer of heterodimers). (**C**). The N-terminus of chimeric type galectin, represented by Gal-3, occurs as a 120-amino acid non-lectin domain attached to a CRD. In addition to the typical, predominant monomeric form in solutions, Gal-3 exhibits a unique ability to form pentamers and dynamic oligomeric lattices, especially on cell surfaces. CRD—carbohydrate recognition domain; C-CRD, N-CRD—C- and N-terminal carbohydrate recognition domains, respectively.

The general division of individual galectins according to their structure is presented in Figure 1.

#### Structure and Spatial Organization of the CRD

The CRD plays a key role in extracellular interactions at the cell surface, including immune modulation [13,89]. Binding of β-galactosides to immune cells and pathogens allows for the control of T-cell activation or apoptosis (e.g., Gal-1 induces T-cell death for immune evasion) and the modulation of innate immune responses [e.g., Gal-3 triggering inflammation through interaction with toll-like receptors (TLRs)] [89,90]. By influencing immune cell migration and shaping inflammatory signals, the CRD has a wide range of effects, including both immunosuppressive (e.g., tumor growth) and inflammatory (e.g., autoimmunity) effects [91,92].

The galectin CRD is composed of approximately 130–140 amino acid residues that form a two-layered, antiparallel β-sheet sandwich with the shape of a closing hand [81]. The back part (backhand) is formed by threads from F1 to F5 (forming the F-sheet), whereas “the grip part” (palm) consists of threads from S1 to S6 (S-sheet). In all galectins, the carbohydrate-binding site (CBS) is located in the groove on the S-sheet side of the sandwich, and the β-galactoside recognition core motif is mediated by the S4, S5, and S6 threads [81,93]. The CRD fold structure is highly conserved, as evidenced by the fact that the maximum backbone root mean square deviation (RMSD) between all human galectins is less than 2.2 Å, with the main differences observed in specific loop regions [81,94].

**Figure 2:**
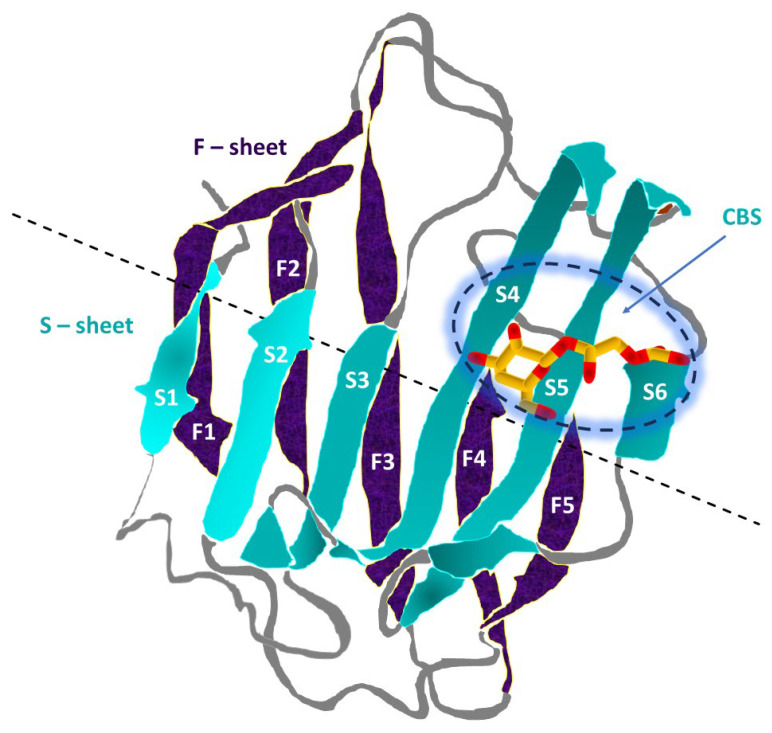
General structure of the conserved carbohydrate recognition domain (CRD) of galectins. A chain of approximately 130–140 amino acids folding into a characteristic β-sandwich with two antiparallel sheets, creates a shape resembling a “closing hand”. The back of this hand, consisting of strands (threads) from F1 to F5, forms the F-sheet, while the palmar surface (“the grip part”) is composed of strands from S1 to S6 (S-sheet). In all galectins, the sugar binding site or carbohydrate binding site (CBS) is located in a groove on the S-sheet, where key residues in S4, S5 and S6 threads mediate binding to β-galactosides, forming a core recognition motif.

The structure and spatial organization of the CRD are outlined in Figure 2.

### 2.2. Key Features of Galectin Signaling

As glycan often accounts for more than half of the molecular weight of many glycoproteins and nearly 80% of all human proteins possess glycan modifications, glycans provide a very unique and highly regulatable substrate for glycan-binding proteins (GBPs), including β-galactose-containing glycans, which are substrates for galectins, to interact with cells [95]. Moreover, with few exceptions, cell surface receptors and secreted proteins are heavily glycosylated [96].

#### 2.2.1. Galectins as “Molecular Matchmakers”

Galectins act as receptors or ligands for various glycoproteins and glycolipids, and signaling associated with them involves the binding of these glycans to the cell surface or the activation of pathways toward intracellular targets [2,11].

The recognition and binding of β-galactosides by galectins are supported by many receptors, including T-cell receptors (TCRs), B-cell receptors (BCRs), integrins, mucins, sialic acid-binding lectins (Siglecs), immune checkpoint receptors [T-cell immunoglobulin and mucin domain-containing-3 (TIM-3), programmed cell death protein 1 (PD-1), and lymphocyte-activated gene-3 (LAG3) protein], the main receptors that induce a proinflammatory and anti-inflammatory microglial phenotype [toll-like receptor 4 (TLR4)/triggering receptor expressed on myeloid cells 2 (TREM2), respectively], cytokine receptors, and receptor tyrosine kinases [e.g., vascular endothelial growth factor receptor 2 (VEGFR2)] [13,97]. By forming lattices of receptors or signaling platforms that modulate cell signaling, as well as through cytokine scavenging/modulation and intracellular protein interactions, galectins significantly influence various functions, such as cell migration and adhesion, immune responses, immune evasion in cancer and cell survival or apoptosis [13,98,99]. In addition to immune checkpoint receptors that can co-mediate at least some galectin functions, galectins act as pattern recognition receptors (PRRs) for pathogens and intracellular targets such as Bcl-2 [62,100,101,102].

Given that some members of the galectin family may act as resolution-associated molecular patterns (RAMPs) and pathogen-associated molecular patterns (DAMPs), the impact of the surrounding environment profoundly defines whether the inflammatory response becomes dampened, depending on the interplay between anti-inflammatory and proresolving mediators, or amplified by a predominance of proinflammatory mediators [103,104].

#### 2.2.2. Interaction of Galectins with Intracellular Vesicles

Acting as tiny transport bubbles that move substances such as proteins, lipids, and hormones within the cell (e.g., to the surface for secretion) or to other organelles (such as lysosomes), intracellular vesicles are crucial for cellular life [105,106]. By organizing cellular trafficking, enabling metabolism, storing substances, or providing a specific form of intercellular communication, intracellular vesicles provide the foundation for cell growth and maintenance of structure and function (e.g., nerve signaling or waste disposal), as well as processes that occur in response to pathogens [47,106,107]. GlcNAc is a key amino sugar that serves as a building block for various glycoconjugates found in both intracellular and extracellular vesicles (EVs) [108,109,110].

Galectins interact with intracellular vesicles primarily as “sentinels” for the integrity of endocytic and lysosomal vesicles and as regulators of trafficking [111,112,113]. Because galectins are synthesized in the cytosol, they are normally separated from their ligands (glycans), which are sequestered inside the lumen of vesicles [113]. Once the membrane of an endocytic vesicle (e.g., endosome, lysosome, or phagosome) is damaged by a pathogen, glycans from the lumen become accessible to galectins in the cytosol. In particular, Gal-3, Gal-8 and Gal-9 can rapidly recognize and bind these newly exposed host β-galactoside-containing glycoconjugates [114]. After binding to the damaged vesicle membrane, galectins deliver “danger signals” that initiate the recruitment of autophagy receptors such as sequestosome 1 (p62), nuclear dot protein 52 kDa (NDP52), or tripartite motif-containing protein 16 (TRIM16) [6]. For example, Gal-3 coordinates lysosomal repair or targets severely damaged vesicles for lysophagy (selective autophagy), whereas Gal-8 can trigger autophagy by recruiting the cargo receptor NDP52 to the damaged site [115]. In this way, the autophagic machinery is activated to sequester the damaged organelle or invading pathogen into an autophagosome for degradation, which is crucial for maintaining cellular homeostasis and antimicrobial defense [116,117].

Galectins, namely, Gal-3, Gal-4 and Gal-9, also play active roles in the recycling and sorting of glycoproteins into specific apical transport vesicles from the trans-Golgi network to the plasma membrane [113,118,119]. Galectin-3 has been found in small GTPase Ras-related protein Rab11 (Rab11)-positive recycling endosomes, where it helps guide the return of specific receptors and carriers to the cell surface [120,121].

#### 2.2.3. Galectins and EVs

Cells release various types of membrane vesicles of endosomal origin (exosomes) and those derived from the plasma membrane (microvesicles) into the extracellular space [122,123]. As carriers of membrane and cytosolic proteins, as well as lipids and RNA, EVs are important links in intercellular communication and participate in the transfer of these substances [124].

Studies on EVs have shown that Gal-1 and Gal-3 can be translocated from the cytosol to the outer cell surface and into the extracellular matrix through microvesicle blebbing [125,126]. Exosome studies have shown that galectins (e.g., Gal-1, Gal-3 and Gal-9) may undergo intracellular recruitment at the intraluminal vesicle (ILV) stage and are precursors of exosomes [127]. Differential Gal-3 expression has been detected in exosomes from many cells, including syncytiotrophoblasts (STBs), macrophages, dendritic cells and stromal cells, with abnormally elevated Gal-3 expression in cells of various neoplasms, including melanoma and cancers of the stomach, liver, pancreas, bladder, ovary, thyroid, and colon [128,129,130,131,132]. Therefore, alterations in exosomal Gal-3 concentrations caused by upregulated Gal-3 recruitment to ILVs may serve as biomarkers for distinct disease states [133,134].

In endometrial small extracellular vesicles (sEVs) that mediate intercellular communication by transporting microRNAs and proteins, the presence of Gal-3 during the implantation phase promotes CTB differentiation into the STB [135]. During this process, Gal-3 on the surface of EVs has been shown to facilitate EV uptake by target cells through clathrin-independent endocytosis using clathrin-independent carriers (CLICs) [135,136]. Among these CLICs, cytoplasmic dynein plays an important role in Gal-3-mediated EV uptake, a minus-end-directed microtubule carrier or motor protein with numerous functions during interphase and mitosis [137].

Gal-1 is highly expressed on the surface of early-gestation chorionic villous-derived placenta mesenchymal stromal cells (PMSCs) and PMSC-derived exosomes. Analysis of the PMSC-derived exosome content revealed the presence of several proteins and RNAs involved in neuronal survival and development. The neuroprotective effect of PMSC-derived exosomes requires the binding of Gal-1 to neural cells, as demonstrated by incubating PMSC-derived exosomes with anti-Gal-1 antibodies [138].

Increased Gal-1 expression in EVs is often associated with immune suppression in the tumor environment but also accompanies the establishment of immunological tolerance during pregnancy at the maternal–fetal interface [13,54,139,140].

The content of Gal-9 in human intestinal epithelial cell (IEC)-derived exosomes contributes to the immunomodulatory effects promoted by most commonly used prebiotics, such as 2′-fucosyllactose (2′FL) and short-chain galacto- and long-chain fructo-oligosaccharides [141]. After exposure to these nondigestible oligosaccharides (NDOs), significantly increased T helper 1 (Th1)-type interferon gamma (IFNγ) levels were observed with concomitant regulatory Gal-9 secretion from IECs [141]. These findings suggest that Gal-9-expressing IEC-derived exosomes play a key role in the mucosal immune regulation induced by NDOs.

Recently, Gal-10 in serum EVs has been included among novel biomarkers (BMs) reflecting disease pathophysiology in bronchial asthma and may represent a new target for liquid biopsy approaches. During the validation of BMs, it was determined that the potential of Gal-10 in EVs was superior to that in eosinophils in terms of diagnostic capability and detection of airway obstruction [142].

#### 2.2.4. Nonclassical Secretion of Galectins

The classical transport pathway starts at the rough endoplasmic reticulum (ER) and is followed by vesicular transport to the Golgi apparatus and then to the cell surface via secretory follicles; this process involves synthesis on ER ribosomes, transport to the Golgi for modification/sorting, packaging into vesicles budding from the trans-Golgi network (TGN), and, finally, fusion with the plasma membrane to release contents [143,144].

Galectins are secreted through so-called nonclassical or unconventional protein secretion (UPS) pathways, which include exosomes or lysosomes, allowing them to function both inside (cytoplasm, nucleus) and outside (cell surface, extracellular matrix) the cell [113,120,145,146]. Galectins are synthesized on cytosolic free ribosomes and remain in the cytoplasm, bypassing the ER and Golgi apparatus, where glycosylation normally occurs [26]. Therefore, the unconventional release mechanism explains why galectins are typically nonglycosylated, unlike proteins that follow the standard route [145].

**Figure 3:**
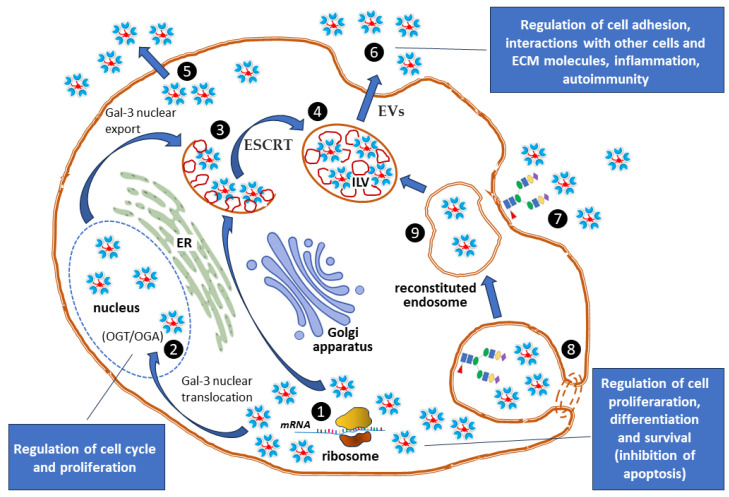
Non-classical secretion or unconventional protein secretion (UPS) pathway for galectine-3 (Gal-3). Once synthesized on cytoplasmic free ribosomes, Gal-3 remains in the cytoplasm, bypassing the endoplasmic reticulum (ER) and the Golgi apparatus ❶. Translocation of cytoplasmic Gal-3 to the cell nucleus enables O-GlcNAcylation, the degree of which depends on the functional balance between enzymes with opposing effects; i.e., O-GlcNAc transferase (OGT) responsible for the attachment of a single N-acetylglucosamine (GlcNAc) moiety and O-GlcNAcase (OGA) catalyzing the removal of the O-GlcNAc moiety from modified proteins through hydrolysis ❷. Both O-GlcNAcylation and deglycosylation are necessary for the proper functioning of the Gal-3 secretion process. Subsequently, after leaving the nucleus, apical sorting ❸ enables selective packaging of Gal-3 into intraluminal vesicles (ILVs) ❹ via the endosomal complex required for transport (ESCRT). Some of the Gal-3 exported from the nucleus may undergo direct translocation across the membrane (in vitro data) using interactions with plasma membrane lipids ❺. ILVs are released into the extracellular space in the process of exocytosis, after fusion of extracellular vesicles (EVs) with the cell’s plasma membrane ❻. Glycan-mediated Gal-3 recognition occurs at the cell membrane surface ❼, which may be followed by internalization of Gal-3 through clathrin-independent endocytosis (CIE) ❽. Thus, a specific recycling of Gal-3 is ensured inside the cell ❾. The text in the blue boxes summarizes the main actions of Gal-3 in the cytoplasm, nucleus, and on the outer surface of the cell membrane and in the extracellular matrix (ECM), respectively.

An example of a nonclassical secretion pathway in the case of Gal-3 is the sorting of cargo by members of the endosomal complex required for transport (ESCRT) into ILVs at the membrane of multivesicular endosomes (MVEs) [147]. Gal-3 interacts in the cytosol with tumor susceptibility gene 101 (TSG101), a component of the ESCRT-I complex [148]. Such direct interaction, facilitated by a highly conserved tetrapeptide motif P(S/T)AP in the amino-terminal domain of Gal-3, ensures its recruitment into ILVs. ILVs are released into the extracellular space as EVs (exosomes) when multivesicular bodies (MVBs), which contain ILVs, fuse with the plasma membrane of the cell [149]. During this process, the ESCRT machinery is assisted by Rab GTPases, a large family of small proteins that act as master regulators of all intracellular vesicle trafficking, controlling steps such as vesicle budding, movement, docking, and fusion. Rabs act as on/off switches, being active when bound to guanosine triphosphate (GTP) and inactive when bound to guanosine diphosphate (GDP). In addition, the ability of Gal-3 to interact with membrane lipids suggests the possibility of spontaneous penetration of lipid bilayers [150]. The non-classical secretion pathway for Gal-3 is presented in Figure 3.

Identification of the specific, nonclassical secretion of other galectin family members present in EVs, such as Gal-1, Gal-4, Gal-9, Gal-10 and Gal-13, requires further clarification [141,151,152,153]. For example, Gal-10 is released by eosinophils during a specific form of active cell death called eosinophil extracellular trap cell death (EETosis), where it exits the cell along with DNA nets (EETs) and granules, often forming Charcot–Leyden crystals (CLCs) and acting as a biomarker for eosinophilic diseases such as eosinophilic esophagitis (EoE) and eosinophilic granulomatosis with polyangiitis (EGPA) [154]. This nonclassical secretion of Gal-10 involves plasma membrane rupture, making it a key indicator of eosinophil activation and inflammation in tissues because lytic degranulation of eosinophils also elevates serum levels of galectin-10 [155].

The interaction of Gal-13 [also known as placental protein 13 (PP13)] from placental tissue and fetal hepatic cells with cytosolic annexin II at the plasma membrane plays an important role in the nonclassical secretion of this lectin. Annexin II-dependent Gal-13 secretion may have special hemostatic and immunobiological functions at the lining of common feto-maternal blood spaces or a developmental role in the placenta [76,145]. Gal-13 could be colocalized with annexin II and actin in syncytiotrophoblasts. It has been proposed that the presence of Gal-13 in the appropriate time window during pregnancy facilitates the expansion of uterine arteries and veins in an endothelial cell-dependent manner via the endothelial nitric oxide synthase (eNOS) and prostaglandin signaling pathways [35,156].

#### 2.2.5. Galectins in Angiogenesis

Angiogenesis is a vital biological process through which new blood vessels form from preexisting vessels through sprouting and remodeling [157]. Such expansion of the vasculature is essential for normal functions such as wound healing and development, but in many cases, it plays a key role in disease progression, especially in cancer development (tumorigenesis) and metastasis [158,159,160]. Pathologic angiogenesis is also a hallmark of diabetes, where poor glycemic control with chronic hyperglycemia leads to metabolic imbalance, resulting in both excessive (as in diabetic retinopathy) and defective (as in diabetic foot wound healing) vessel growth [161,162,163].

During angiogenesis, which is a complex and tightly regulated process, endothelial cells (ECs) that form the lining of vessels adopt different phenotypes, which allow them to proliferate, migrate, and form tube-like structures, the precursors of new vessels [164]. Such transformations require the interaction of two cell surface glycans and, consequently, endothelial galectin expression, as has been conclusively demonstrated in studies on placental angiogenesis [165,166]. Among the members of the galectin family, Gal-1, Gal-3, Gal-8 and Gal-9 have been shown to be particularly important as key regulators of ECs during angiogenesis, influencing its main pathways related to, among other processes, vascular endothelial growth factor (VEGF)/VEGFR2 and neurogenic locus notch homolog protein (Notch) signaling [15,167,168].

Gal-1 is involved in the regulation of alternative splicing patterns for genes related to angiogenesis, especially those controlling focal adhesion and angiogenesis-associated VEGF signaling. By binding to RNA transcripts, lectins can influence the transcriptome profiles of human umbilical vein endothelial cells (HUVECs), as confirmed by the significantly impaired expression of angiogenesis-related genes in the case of Gal-1 knockdown [167,169].

Like Gal-1, Gal-2 stimulates phosphorylation and signaling via VEGF2 [167,170]. By “turning on” the expression of Jagged-1 (JAG1), a critical Notch ligand in ECs, Gal-3 acts as an angiogenic factor and a primary regulator of vascular development, maturation, and homeostasis [168]. However, in the presence of oxidized low-density lipoprotein, Gal-3 has been shown to inhibit endothelial cell proliferation and exacerbate EC dysfunction [171,172].

Tandem repeat-type Gal-8 is an important component of the angiogenesis network in both blood vessels and lymphatic vessels. Gal-8 controls capillary tube formation and EC migration through the use of the following endothelial ligands: activated leukocyte cell adhesion molecule (ALCAM, also known as CD166) in ECs and podoplanin (PDPN) in lymphatic vessels [173]. Gal-8 can also bind to VEGFR2 and increase endothelial permeability through the eNOS pathway with S-nitrosylation-dependent adherens junction disassembly [174].

As demonstrated in studies using a Matrigel plug experiment, Gal-9, particularly its dominant isoform in endothelial cells, galectin-9M, serves as a chemoattractant for ECs, while chicken chorioallantoic membrane (CAM) assays confirmed the inhibitory effects of Gal-9M on EC proliferation and migration [175]. However, further studies on the role of Gal-9 in angiogenesis are necessary, especially those considering the concentration-dependent and biphasic effects of galectin-9M, the presence of multiple Gal-9 isoforms under natural conditions, and the susceptibility of Gal-9 to proteolytic cleavage, cellular activation status, and cell origin, as primary ECs are more sensitive than immortalized ECs are [175,176].

Notably, the mRNA expression levels of three other galectins (Gal-2, Gal-4 and Gal-12) in normal ECs are low and have often not been confirmed at the protein level, and mRNA expression of the remaining galectins (Gal-7, Gal-10, Gal 13 and Gal-14) is not detected at all [165]. However, the expression of various galectins may be altered in endothelial dysfunction associated with chronic low-grade inflammation, as demonstrated by the upregulated, strong expression of Gal-2 in extravillous trophoblasts (EVTs) and fetal ECs in GDM [177]. The cellular context, including the functional state of the cell, may be important for specific endothelial galectin expression, as indicated by greater Gal-8 expression in primary isolated lymphatic ECs than in regular ECs [178,179]. Similarly, Gal-3 expression appears to be greater in endothelial progenitor cells than in normal ECs [180].

Moreover, the differential expression of angiogenesis-related galectins is influenced by numerous cytokines, which requires further systematic study. For example, Gal-1 expression is induced by interleukin-1 beta (IL-1β), IFNγ, tumor necrosis factor alpha (TNFα), low-density lipoproteins (LDLs), lipopolysaccharide (LPS), and cathepsin L; increased Gal-3 expression may be due to the action of IL-1β, fibronectin, advanced glycosylation end products (AGEs), asialofetuin, and neutrophil adhesion/transmigration; and Gal-9 expression is induced by IFNγ, IFNβ, IL-10, and viral RNA [165].

## 3. The Expression and Importance of Galectins at the Maternal–Fetal Interface and in the Placenta

Galectins play extremely important roles in both the male and female reproductive systems [22,26,181], especially in the latter, and their role becomes crucial for the course of almost all phenomena related to reproduction, from fertilization of the egg [182,183] and implantation of the blastocyst [184], with the establishment of immune tolerance [20,21], through placentation and regulation of angiogenesis during placental development [185,186,187,188], to the occurrence of uterine contractions and labor [189,190].

The maternal–fetal interface encompasses all anatomical sites where maternal and fetal tissues interact to support a healthy pregnancy. It is primarily composed of two main compartments: the decidua on the maternal site and trophoblasts (STBs and CTBs), creating the complex structure of the placenta and membranes on the fetal side [191].

Notably, the expression of all galectins in humans has been demonstrated at the maternal–fetal interface (see Table 1, the text marked in bold), with the strength of expression of individual galectins and its variability over time strongly depending on the stage/phase of pregnancy. Developmentally regulated and trophoblast differentiation-dependent expression patterns are particularly associated with Gal-1, Gal-3, Gal-8, Gal-13 and Gal-14 [166]. Galectins that are exclusively expressed in the placenta include Gal-13 and Gal-16, whereas Gal-14 has been shown to be expressed outside the placenta only in eosinophils [19,45,79]. These galectins induce apoptosis in activated T cells and polarize neutrophils toward an immunoregulatory phenotype to protect the embryo from the mother’s immune system [21].

Maternal and placental galectins pass (probably by leakage) into the maternal circulatory system; for example, soluble Gal-1 and Gal-13 concentrations in blood increase steadily from the first to the third trimester but then disappear or decrease rapidly during the postpartum period [22,192,193]. The diagnostic importance of monitoring blood levels of individual galectins during pregnancy (e.g., Gal-1, Gal-3, Gal-9 and Gal-13) is the subject of ongoing research that requires large-scale validation to become routine in clinical practice [22].

Gal-1, Gal-2 and Gal-3 are particularly expressed and widely distributed in the endometrium, decidua and placenta (EVTs, STBs), and Gal-1 is additionally expressed in EVT immune cells, including decidual NK cells and CD4^+^CD25^+^ Tregs. Therefore, Gal-1 has important immunological functions, including estrogen-mediated regulation of Gal-1 in maternal–fetal immune tolerance during implantation and placentation [140,194]. Gal-1 is a potent inducer of apoptosis in activated CD8^+^ (Th1) and CD4^+^ (Th17) T cells. Gal-1 acts by binding to specific glycoproteins such as CD7, CD43, and CD45 on the T-cell surface [139]. Gal-2 is primarily immunomodulatory, exerting anti-inflammatory effects [58].

The presence of Gal-3, a protein that is upregulated during the implantation phase and known to be involved in CTB cell fusion and differentiation into STBs, in endometrial sEVs also increases endometrial (decidual) receptivity, promoting blastocyst implantation [52,58,135]. With respect to apoptosis, Gal-3 exhibits functional dualism. While intracellular Gal-3 can inhibit programmed cell death, extracellular Gal-3 induces T-cell apoptosis by binding to receptors such as CD7, CD29 and CD71 [195].

Gal-1 and Gal-3 promote the migratory and invasive phenotype of EVTs, which is critical for remodeling maternal spiral arteries to ensure adequate blood flow to the placenta [196,197]. Consequently, deficiencies in Gal-1 and Gal-3 are associated with placental malperfusion and inflammation, leading to fetal growth restriction (FGR) [197,198,199,200].

Gal-4 expressed in the endometrium is crucial for placentation and acts as a regulator of trophoblast differentiation and adhesion, with its levels decreasing as trophoblasts mature, suggesting that it plays a role in establishing the maternal–fetal interface by controlling cell migration and potentially influencing inflammation [52,201].

The actions of Gal-7, which is expressed in the decidua, endometrium, EVTs, and glandular epithelial cells, are largely associated with cell adhesion, namely, with facilitating trophoblast–endometrial epithelial cell adhesion [52,56]. The results of studies on Gal-7 expression in placental tissue suggest that its increased concentrations (also in serum) may be the result of oxidative stress, including that accompanying preeclampsia and GDM. Increased Gal-7 production was demonstrated in chorionic villous samples from women who subsequently developed preterm preeclampsia compared with those from women with uncomplicated pregnancies. In vivo, upregulated Gal-7 expression led to impaired human first-trimester trophoblast outgrowth, increased placental production of the antiangiogenic soluble fms-like tyrosine kinase-1 (sFlt-1 or sVEGFR1) splice variant sFlt-1-e15a, and reduced placental production and secretion of angiogenesis promoters, such as a disintegrin and metalloprotease 12 (ADAM12) and angiotensinogen [202]. Gal-7 administered to pregnant mice caused hypertension and albuminuria associated with dysregulation of the renin–angiotensin system (RAS) and impaired placentation with reduced labyrinth vascular branching, impaired decidual spiral artery remodeling, and a proinflammatory placental state with elevated IL1β and IL6 levels and reduced IL10 levels [39,202,203].

Gal-8 is expressed in the human placenta mainly in EVTs and VTs [24,52]. Under physiological conditions, Gal-8 promotes trophoblast invasion by increasing the expression and activity of matrix metalloproteinases (MMPs), particularly MMP-2 and MMP-9, and can mediate cell–cell interactions, potentially impacting pregnancy complications such as preeclampsia by affecting trophoblast invasion and uterine remodeling [24,69]. Gal-8 also induces apoptosis in activated T cells through the intrinsic mitochondrial pathway involving caspase-3 and caspase-9 activation [204].

Gal-9, like Gal-1 and Gal-3, is highly expressed in the endometrial epithelium during the “window of implantation,” facilitating the initial attachment of the embryo to the decidua. Gal-9 and TIM-3 form an immune checkpoint pathway that is crucial for maternal–fetal immune tolerance, where Gal-9 binds to TIM-3 on immune cells, often leading to immune suppression and T-cell exhaustion characterized by the stepwise and progressive loss of T-cell function [52,70].

Gal-10 is a cytoplasmic protein of human eosinophils and is involved in various eosinophilic diseases [155]. Studies on placentas from pregnancies complicated by GDM have shown that the increased inflammatory response in placental tissue is accompanied by increased Gal-10 expression [72].

Gal-12, an essential protein because of its influence on lipolysis and inflammatory processes, is expressed in the endometrium and in STBs and EVTs [41,52,73]. This expression is significantly upregulated in placentas from women with GDM compared with placentas from healthy controls, particularly in the nuclei of STBs and EVTs, which may confirm the association of Gal-12 with the formation of a proinflammatory background during the course of diabetes [41].

Gal-13 was cloned from the human placenta as PP13 and later classified as a member of the galectin family [22]. Gal-13 facilitates the remodeling and expansion of uterine arteries and veins during pregnancy in an endothelial cell-dependent manner via the eNOS and prostaglandin (PGE_2_) signaling pathways [35,205]. In this way, Gal-13 ensures that the optimal amount of blood, oxygen, and nutrients reaches the placenta and the developing fetus. Reduced levels of Gal-13, as well as Gal-1 and Gal-14, have been described in patients with defective spiral artery remodeling manifesting as preeclampsia. Therefore, profiling of galectin levels (especially Gal-1 and Gal-3) in maternal serum is currently being actively evaluated in the search for biomarkers that can serve as predictive diagnostic tools for the early detection of preeclampsia [206,207,208].

Predominantly expressed in STBs, a multinucleate, terminally differentiated syncytium, prototype Gal-14 is another representative galectin whose placental expression is developmentally regulated and dependent on trophoblast differentiation [20,74,209]. Given that Gal-14 expression in the human placenta exceeds the expression of other galectins, its role in fetal development and the regulation of immune tolerance may be dominant. Low expression of Gal-14, as well as Gal-1 and Gal-9, is frequently reported in cases of spontaneous and recurrent pregnancy loss [19,77].

Labeling by fusion with enhanced green fluorescent protein (EGFP) revealed the localization of EGFP-tagged Gal-14 primarily in the cell nucleus, where it colocalized with the proto-oncogene c-Rel, a key transcription factor in the nuclear factor kappa-light-chain-enhancer of activated B cells (NF-κB) family [77,210]. These findings suggest that Gal-14 signaling may, at least in part, be mediated through NF-κB-related pathways.

Gal-16 is predominantly and highly expressed in the placenta, where it is localized mainly in differentiated trophoblasts, STBs and the endothelium of fetal vessels [19,74,80]. It has been shown in two placental cell lines (BeWo and JEG-3) that trophoblast differentiation is accompanied by upregulated expression of *LGALS16* [80]. An important function of Gal-16 as well as placental Gal-13 and Gal-14 is the ability to induce apoptosis in CD3^+^ T cells, which may contribute significantly to immune tolerance at the maternal–fetal interface [21,210]. Like Gal-14, intracellular EGFP-trapped recombinant Gal-16 was localized predominantly in the nucleus of transfected cells, including HeLa, 293T, HCT-116, SMMC-7721 and Jurkat cells, in colocalization with c-Rel [210,211]. Processes such as inflammation, apoptosis, cell growth and differentiation, and the immune response in the placental compartment may therefore be mediated by NF-κB-dependent signaling pathways [210,212].

Analysis of the expression patterns of galectin family members, such as the members involved in angiogenesis in the placenta (Gal-1, Gal-3, Gal-8 and Gal-9), may be important not only for understanding their physiological significance but also for determining the involvement of this class of lectins in the pathomechanisms of pregnancy complications [19,213].

**Figure 4:**
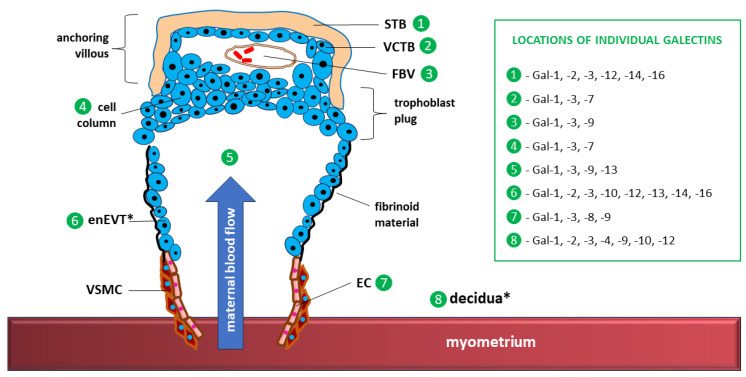
The expression pattern of galectins at the level of the maternal–fetal interface and placenta. The strength of this expression and localization of galectins are clearly developmentally regulated and dependent on trophoblast differentiation [9,19,20,26,45,46,74,214]. EC—endothelial cell, enEVT—endovascular extravillous trophoblast, STB—syncytiotrophoblast, VCTB—villous cytotrophoblast, VSMC—vascular smooth muscle cell, FBV—fetal blood vessel, * including immune cells.

The expression of individual galectins at specific locations within the maternal–fetal interface and forming placenta is shown in Figure 4.

Since the presence of galectins in maternal–fetal tissues plays a central role in a successful pregnancy by regulating immune tolerance, angiogenesis, and trophoblast invasion, the dysregulation of these proteins often leads to “great obstetrical syndromes” and various adverse perinatal outcomes, including fetal growth restriction, GDM, early-onset preeclampsia and a spontaneous miscarriage [21,22,36,45,140].

## 4. Galectins and Placental Dysfunction in GDM

The terms “placental dysfunction” or “placental insufficiency” cover a wide spectrum of obstetric complications caused by insufficient transfer of oxygen and nutrients to the fetus via the placenta [215,216]. Typically, placental function gradually deteriorates, and fetal hypoxia may cause fetal growth retardation/restriction (FGR) [217,218]. However, disorders in GDM create a very complex environment for pregnancy development, which causes mainly fetal macrosomia (excessive growth), despite placental issues, rather than FGR [219,220,221]. Macrosomia is the result of excess glucose passing through the placenta to the fetus, causing fetal hyperglycemia and increased insulin production by the fetal pancreas with subsequent accumulation of fat and protein. Fetal macrosomia, defined as a birth weight ≥ 4000 g, may affect 12% of newborns of normal women and 15–45% of newborns of women with GDM [222]. Less frequently, in approximately 8–12% of cases, according to various estimates, microvascular damage in the placenta during severe GDM can impair the delivery of oxygen, amino acids and other nutrients, limiting fetal growth [223,224,225]. Nutritional imbalance with both excessive nutrient delivery (glucose) and insufficient delivery (other essential nutrients) creates conflicting growth signals. Therefore, depending on the severity and duration of hyperglycemia in GDM, both outcomes are possible: macrosomia and FGR [221]. Changes in placental morphology and function, and therefore the frequency and severity of complications, are inversely proportional to the degree of glycemic control (balance) after the diagnosis of GDM [226,227].

### 4.1. Abnormal Placental Morphology in GDM

Ultrastructural evaluation of the placenta in GDM for anatomical changes associated with impaired glucose metabolism and its consequences is typically performed during the third trimester of pregnancy, most often after delivery [228], when microscopic and macroscopic alterations in placental anatomy and function are then fully developed [228,229]. Changes in the placenta are primarily adaptive or compensatory to maternal hyperglycemia, but as pregnancy progresses, after exceeding the ability to counteract hypoxia, oxidative stress and the developing inflammatory environment, these changes create the anatomical basis for functional deficits and complications in the fetus/newborn [230,231,232].

The ultrastructural characteristics of terminal villi in GDM pregnancies differ from those in placentas obtained after normal pregnancies [233]. In comparative studies of the morphology of the maternal–fetal interface of the diabetic placenta using light microscopy (LM) and transmission electron microscopy (TEM), significantly more frequent degenerative changes in the terminal villi, thickening of the basal membrane (BM) of the vasculosyncytial membrane (VSM) and the VSM itself were noted; these changes significantly reduced the number of or even caused the absence of STB microvilli, caused the swelling or even complete destruction of mitochondria (shape irregularities) and dilation of rough and smooth endoplasmic reticula (ERs), and caused STBs to be interspersed with multiple vacuoles. In some areas of microscopically examined specimens of GDM placentas, a complete absence of microvilli was revealed [234,235,236,237].

Studies on the macroscopic morphology and histoarchitecture of placentas in full-term pregnant women with gestational diabetes have shown increased weight, thickness, decidual vasculopathy and retroplacental hemorrhage, villous enlargement (edema) and agglutination, along with features such as syncytial knotting, villous fibrinoid necrosis, calcification, increased collagen, and a form of hypervascularization called chorangiosis, with an excessive number of blood vessels (capillaries) in the terminal villi [238].

In a study assessing the incidence of placental vasculopathies in GDM, the most common placental changes on the maternal side of the placenta were syncytial knots (77%), calcification (70%), villous agglutination (57%), decidual vasculopathy (43%), and retroplacental hemorrhage (34%), whereas on the fetal side, the most common vasculopathies included villous fibrinoid necrosis (70.5%), chorangiosis (50%), fibromuscular sclerosis (50%), and villous edema (38.6%) [239].

In placental endothelial cells and in HUVECs, genes involved in signal transduction [like nuclear factor of activated T cells (NFAT), Notch/VEGF], transcription [NF-κB, forkhead box protein O1 (FOXO1)] and mitosis are upregulated, along with increased basal iNOS activity and impaired sensitivity to insulin [231,240,241].

Increased placental angiogenesis is accompanied by increased and altered VEGF expression, reported as either increased or decreased expression of VEGFRs and insulin receptors, probably reflecting the severity of metabolic dysregulation [242,243,244]. Proinflammatory processes in placental tissue, including those mediated by the chemokine fractalkine (chemokine CX3CL1)/fractalkine receptor (CX3CR1) signaling axis, additionally stimulate angiogenesis, although often with an abnormal course [245].

More advanced forms of GDM are accompanied by fibromuscular sclerosis, which refers to thickening and hardening of the placental blood vessel walls [239]. In addition to typical vascular lesions, compared with those in uncomplicated pregnancies, placental morphology in GDM is characterized by “villous immaturity,” which manifests as increased capillaries, altered junctions, and excess leakiness, ultimately impairing nutrient/gas exchange and leading to placental dysfunction [238,239].

### 4.2. Functional Alterations in the GDM Placenta

Structural changes in the placenta resulting from GDM translate into functional disorders that tend to increase with the advancement of pregnancy and become more severe with worsening glycemic control in direct proportion to the prolongation of hyperglycemic periods and maternal blood glucose levels [237,246].

The transport of oxygen to the fetus is compromised, which is usually accompanied by a suboptimal increase in the passage of nutrients through the placenta in terms of quality and energy [247,248]. Metabolic changes associated primarily with chronic placental hypoxia trigger a local inflammatory response in response to oxidative stress. Such an inflammatory background indicates increased concentrations of proinflammatory cytokines and inflammatory markers, which exacerbate metabolic disorders [249]. Not only does the endothelium become dysfunctional, but ECs can also lose integrity, normal senescence, and the ability to repair, progressing to cell death, including apoptosis and pyroptosis, and leading to impaired barrier function (endothelial leakage) [250]. Reduced occupancy of junctional occludin is a feature of human placental vessels in the diabetic milieu, along with downregulation of occludin (OCLN) gene variant 2 and the fully functional occluding isoform-A protein [251]. Moreover, dyslipidemia and altered levels of hormones and growth factors, including insulin, insulin-like growth factors (IGFs), placental growth factor (PlGF), human placental lactogen (hPL), leptin and resistin, significantly affect the course of pregnancy [246].

In GDM, many signaling pathways in placental tissue are disrupted or interrupted, including those related to sirtuins, peroxisome proliferator-activated receptors (PPARs), 5′ AMP-activated protein kinase (AMPK), NF-κB, glycogen synthase kinase 3 (GSK-3), inflammasome signaling, mechanistic target of rapamycin (mTOR) and ER stress signaling through the unfolded protein response (UPR) [219,252,253,254,255,256].

During very early pregnancy, in the postimplantation stage, functional changes accompanying impaired glucose tolerance (IGT) and GDM may ultimately lead to changes in EVT invasion, leading to an increased risk of early pregnancy loss, growth retardation and/or preeclampsia [257,258].

Disturbances in trophoblast invasiveness and angiogenesis are accompanied by significant changes in the levels of angiogenic biomarkers in placental samples obtained from GDM pregnancies, with significantly higher levels of VEGF-A, endoglin, endothelin-1 (ET-1), and angiopoietin-2 (Ang2) in these patients than in normal controls [259,260].

It has also been shown that placental dysfunction in diabetic pregnancies with poor glucose control is linked to an increased number of fetal cells (fetal microchimerism or FMc) in the mother’s blood. It has been hypothesized that endothelial leakage facilitates the transfer of fetal cells into the maternal circulation, increasing FMc. The prevalence of FMc is greater in GDM patients with fetal birth weights below the 10th or above the 90th percentile [261].

#### Nutritional Imbalances in Placental Transport

Given that the placenta acts as a nutrient sensor, placental dysfunction in GDM also affects the transport of glucose, amino acids, and lipids [262]. Typically, there is increased transport of glucose and amino acids, leading to excess nutrient delivery that promotes fetal overgrowth (macrosomia) and fat deposition, contributing to long-term metabolic risks for the child. While glucose transfer is often limited by placental blood flow, upregulation of active amino acid transporters such as System A combined with increased lipid transport (especially in the case of visceral obesity in the mother) leads to an excessive supply of energy and building substrates [263]. Interestingly, GDM does not increase placental nutrient transporter abundance when birthweight is within the normal range. In this case, compensatory downregulation of some transporters, together with vascular perfusion, is more likely to affect pregnancy outcomes and metabolic development in the infant [248].

Disturbances in placental glucose transport in GDM are accompanied by complex changes in the expression and activity of individual membrane proteins, including native solute carrier 2A (SLC2A), also known as glucose transporters (GLUTs). For example, increased GLUT-1 expression in trophoblast cells and placental ECs was demonstrated with concomitant upregulation of insulin-dependent GLUT-4, whereas high-affinity transporter GLUT-3 expression was downregulated in GDM. Significant changes in the expression of GLUT-9, which is involved in glucose/fructose transfer, have also been demonstrated, with some studies showing increased expression. Changes in the expression of other GLUT isoforms, such as GLUT-8 and GLUT-12, are linked to fetal size [264,265,266,267].

GDM significantly increases endothelial lipase (EL) expression in the human placenta [268]. This is caused by metabolic inflammation within the maternal–fetal interface with increased leptin activity and local increases in the TNFα concentration. EL is crucial for hydrolyzing maternal lipoprotein lipids (e.g., triglycerides and phospholipids) to release fatty acids, which are then transported to the fetus [269,270,271]. However, in relation to other placental lipid transporters, such as lipoprotein lipase (LPL) and long-chain fatty acid transport protein 6 [SLC27A6, also known as fatty acid transport protein 6 (FATP6)], no differences in placental expression were demonstrated between the well-controlled GDM and control (normal) groups [272].

Placental transport of amino acids is also disturbed in GDM, resulting in increased concentrations of both essential and nonessential amino acids [273]. Both amino acid transport System A within STBs (especially in microvilli), which is responsible for the transport of alanine, glycine, and serine, and amino acid transport System L, which is responsible for the transport of high-molecular-weight amino acids (e.g., leucine), which has been detected in microvilli and on the BM, are upregulated by insulin, leptin, IGF-1, and IL-6 [273,274].

### 4.3. Changes in Placental Galectin Expression and Placental Dysfunction in GDM

Aberrant carbohydrate metabolism in GDM causes significant disruption of glucose homeostasis and lipid metabolism, resulting in various N-glycosylation features [275]. Changes in glycocodes that can be identified with shifts in the placental N-glycome, the ratio of glycoforms to N-glycosylated proteins, or the levels of GBPs required for trophoblast migration and invasion as well as immune modulation are present in GDM [166,276,277]. Several differences in the N-glycosylation pattern between GDM-complicated pregnancies and normal pregnancies can be indirectly demonstrated in placental tissue by examining the expression of individual galectins or using their respective glycobiomarkers [278,279,280,281]. In diabetes complicated by GDM, abnormal glycosylation and activity of the galectin–glycan axis are largely due to an imbalance of placental galectins [26]. However, in most cases, further research is needed to clarify whether the altered expression of a given galectin in placental tissue is the result of homeostatic disturbances accompanying GDM or whether it is itself a pathomechanism of GDM.

With respect to Gal-1, most studies have consistently reported an increase in its concentration in pregnant women’s serum during mid- to late-term pregnancy, which is correlated with Gal-1 overexpression in placental tissue [282,283,284]. Aberrant Gal-1 regulation has been detected in the local and peripheral circulation of the placenta in GDM patients, and the severity of these disorders is related to the severity of glycemic disorders [45]. Another study revealed that during normal pregnancy, the concentration of Gal-1 in serum increased during subsequent weeks of pregnancy, but the pattern of Gal-1 secretion was unchanged [36]. The association found in this study between the galectin-1 5′ regulatory LGALS1 SNP rs4820294 (C/T) gene polymorphism and GDM-complicated pregnancy requires confirmation in a larger group [36]. Increased Gal-1 expression in the diabetic placenta is linked to impaired placental development, altered trophoblast function (invasion/migration), poor spiral artery remodeling, and disturbed maternal–fetal immune tolerance, potentially leading to complications such as fetal growth restriction (FGR), preeclampsia, and adverse pregnancy outcomes by disrupting normal placentation processes and promoting inflammation [22,26,45].

In GDM, placental Gal-2 expression is upregulated, as has been demonstrated in the fetal STB and in the maternal decidua. However, further studies are needed to determine whether the overexpression of Gal-1 is induced by the presence of proinflammatory factors in the surrounding environment or whether Gal-1 itself participates in the pathomechanism promoting the disorders associated with GDM [177].

Data on both placental Gal-3 expression and the serum Gal-3 concentration show significant variability depending on the period (trimester) of pregnancy and may indicate that Gal-3 is sensitive to the hormonal and metabolic changes that characterize GDM [37,285]. Some researchers have suggested that pregnant women with higher serum Gal-3 levels are more likely to develop GDM later in pregnancy than women with lower Gal-3 levels are. These findings may provide a basis for further research into the use of Gal-3 in the first trimester of pregnancy as a potential biomarker for the development of insulin resistance and GDM [37,286,287,288]. Analysis of placental biopsies have confirmed elevated Gal-3 mRNA expression in GDM pregnancies compared with normal pregnancies, accompanied by increased expression of placental Gal-3 protein. Immunohistochemically, upregulated Gal-3 expression in GDM placental EVTs has also been demonstrated [37]. Given that Gal-3 plays important roles in pathological or borderline processes such as inflammation, tumor growth, immune response, apoptosis, tissue repair, fibrosis and cardiac remodeling, intracellular changes in placental EVTs resulting from Gal-3 accumulation during the course of pregnancy are not neutral [56]. The overexpression of Gal-3 is accompanied by oxidative stress, the accumulation of AGEs, and the activation of proapoptotic stress signaling pathways, all of which lead to accelerated placental endothelial/EVT dysfunction and altered vascular damage repair [26,289]. Therefore, significant adverse pregnancy outcomes may be predetermined during the first trimester, when Gal-3 is mainly expressed, and promote critical trophoblast functions through inadequate trophoblast invasion and tube formation, which influence further placental development [285].

Recent studies have shown associations between elevated Gal-4 levels in the blood and diabetes, obesity, and heart failure [290]. In the reproductive system, Gal-4 is clearly expressed in the endometrium, and during pregnancy complicated by GDM, Gal-4 is overexpressed in the decidua and STBs. Using a semiquantitative immunoreactivity score (IRS) to quantify cell staining intensity, significantly increased nuclear and cytoplasmic levels of Gal-4 were demonstrated in these cells [38]. Considering the influence of Gal-4 on trophoblast differentiation, maturation and adhesion, an increase in Gal-4 content at the maternal–fetal interface may impair placentation through the suboptimal control of cell migration and possibly the induction of a chronic local inflammatory response [26,52,201].

Comparative evaluation (GDM vs. normal pregnancy) of Gal-7 expression in placental tissue samples, performed using an IRS, revealed upregulation of this galectin in both the STBs, representing the fetal part of the placenta, and the decidua, representing the maternal part of the placenta [39]. A significant increase in Gal-7 expression in the nucleus and cytoplasm of EVTs was also confirmed by double immunofluorescence staining for cytokeratin 7 (CK7), a marker of EVTs [39]. These data provide additional insights into the potential mechanisms underlying the pathogenesis of GDM. The still insufficiently understood contribution of dysregulated (overexpressed) Gal-7 to the inflammatory and metabolic effects associated with GDM may result from the versality of its actions in the endometrium, decidua, EVTs and glandular epithelial cells [26]. These include local inflammation at the maternal–fetal interface with placental release of antiangiogenic factors and impaired placentation, facilitating trophoblast–endometrial epithelial cell adhesion and related trophoblast outgrowth with increased apoptotic resistance, as well as dysregulation of the placental RAS [52,202,203,291].

Like many other galectins, Gal-8 plays a role in immune tolerance at the maternal–fetal interface (e.g., through the intrinsic mitochondrial pathway involving caspase-3 and caspase-9 activation, which may induce apoptosis in activated T cells), but its specific dysregulation in GDM is still being explored. In the placenta of pregnancies complicated by GDM, changes in Gal-8 expression may be accompanied by inflammation and altered trophoblast behavior because by acting on MMP-2 and MMP-9, Gal-8 promotes trophoblast invasion and placental remodeling [24,69,204].

While plasma Gal-9 levels are elevated during the third trimester in pregnant women with GDM and are positively correlated with placental and newborn weight, making Gal-9 a potential biomarker, data on changes in Gal-9 expression in diabetic placental tissue are unavailable [40]. Disturbances in Gal-9 expression during the “implantation window” may affect endometrial receptivity and influence the development of maternal–fetal immunotolerance via the Gal-9/TIM-3 interaction [52,70].

Immunohistochemical staining revealed increased expression of Gal-10 in the placentas of women with GDM compared with those of women in the control group [72]. While Gal-10 is linked to eosinophil activity, its increased placental expression in GDM suggests a broader role in maternal–fetal immune regulation and inflammation, similar to that of other galectins (such as Gal-1, Gal-9, and Gal-13) [155]. STBs showed Gal-10 overexpression in the nucleus and cytoplasm, whereas Gal-10 expression in the decidua was significant in the cytoplasm only [72]. Numerous Gal-10^+^ immune decidual cells, e.g., eosinophils, contribute significantly to the marked increase in decidual Gal-10 expression in the placentas of women with GDM [72]. Assuming that gestational diabetes mellitus is involved in inflammatory processes, galectin-10 might play a role in the development and maintenance of GDM [155,292].

Considering the involvement of Gal-12 in lipolysis and inflammation, its increased expression in placental tissue in GDM, particularly in the nuclei of STBs and EVTs, may correspond with the increased frequency of inflammatory events reported in association with this pregnancy complication [41,293,294]. In terms of only the nuclei of STBs, a positive correlation of Gal-12 expression with maternal BMI and male sex of the fetus was also established, although this finding requires confirmation in a larger study group [26,41].

In pregnancies complicated by GDM, there is a significant reduction in Gal-13 expression in the placenta, particularly in STBs and EVTs [19,26,34]. This is accompanied by a decrease in the serum Gal-13 concentration, suggesting that Gal-13 may be considered a potential biomarker not only for GDM but also for preeclampsia [207,208]. However, one study demonstrated that fetal macrosomia in women with diabetes, including GDM, can also occur with unchanged serum Gal-13 levels [295]. A link has been demonstrated between reduced placental and serum Gal-13 expression and insufficient trophoblast invasion of uterine spiral arteries, with subsequent impaired remodeling of these vessels and placental dysfunction due to impaired perfusion [19,26]. Inflammatory events also increase, reflecting the insufficient anti-inflammatory activity of the reduced Gal-13 concentration [34].

Exclusively expressed in the placenta, the prototype Gal-14 promotes trophoblast migration and invasion by enhancing the expression of MMP-9 and N-cadherin through protein kinase B (PKB, also known as Akt) phosphorylation [22,296]. Compared with all other galectins, this galectin is expressed at significantly higher levels in the human placenta, predominantly in the nuclei of STBs, which may suggest that Gal-14 plays a leading role in fetal development and the regulation of immune tolerance during pregnancy [77]. The role of Gal-14 in the maternal–fetal interface and in the pathophysiology of GDM remains an active area of investigation, often in conjunction with other galectins such as Gal-1, Gal-3, Gal-4 and Gal-13 [19]. While the specific expression levels in placentas collected after pregnancies complicated by GDM are still being determined, it has been suggested that in the case of insufficient induction of T-cell apoptosis, impaired immune tolerance with chronic low-grade inflammation characteristic of GDM should be expected, which may be associated with reduced Gal-14 expression in the placenta [20,77].

Gal-16, which is highly expressed in the human placenta, is involved in processes related to the immune response, inflammation, apoptosis, cell growth and differentiation [19,80]. Dysfunction of these Gal-16 functions is associated with altered activity of NF-κB-dependent signaling pathways [21,74,297]. Studies to determine placental Gal-16 expression in GDM are ongoing.

The aberrant expression of representative galectins in GDM-complicated pregnancies and the potential consequences are summarized in Table 2. However, in most cases, further research is needed to clarify whether the altered expression of a given galectin in placental tissue is the result of homeostasis disorders accompanying GDM or is an inherent pathomechanism of GDM.

According to the current state of knowledge, altered galectin signaling appears to be both a reason for and a result of gestational diabetes mellitus (GDM), with evidence suggesting a complex, bi-directional role in its pathogenesis. Specifically, elevated levels of certain galectins (like Gal-3) in early pregnancy may act as an early indicator of GDM development, while their ongoing dysfunction contributes to the inflammation and insulin resistance that characterize the condition [26].

Moreover, galectins (e.g., Gal-3) may act as significant indicators and potential mediators of cardiometabolic risk (e.g., abdominal obesity, high blood pressure, elevated blood sugar and dyslipidemia) following delivery, particularly in women who experienced pregnancy complications like preeclampsia (PE) or gestational diabetes mellitus (GDM) [287].

### 4.4. Placental Galectins and Insulin Resistance and Mitochondrial Dysfunction in GDM

Galectins have recently attracted the attention of GDM researchers as key regulators of immunological and metabolic processes in the placenta, directly influencing insulin resistance [38,40,298]. In the course of GDM, there is a characteristic dysregulation of many galectins (e.g., circulating Gal-1 deficiency with overexpression in placental tissue, increased expression of Gal-3 in blood and placenta, decreased placental expression of Gal-9 and Gal-13; see also Section 4.3), which promotes the development of chronic low-grade inflammation within the uteroplacental unit [34,40,299].

One of the best known markers of insulin resistance is Gal-3, the elevated level of which correlates with higher progesterone levels and the severity of glucose intolerance, primarily due to its stimulating effect on the mitogen-activated protein kinase (MAPK) pathways, including Janus kinases (JNKs), p38 mitogen-activated protein kinase (p38) and extracellular signal-regulated kinase (ERK), and not insulin secretion itself [288,299]. A strong correlation was demonstrated between elevated Gal-3 levels in the serum of pregnant women with GDM and the homeostatic model assessment—insulin resistance (HOMA-IR) index, which assesses the ratio between glucose and insulin concentrations [298]. Acting as a mediator, Gal-3 directly promotes insulin resistance by inducing inflammation in adipose tissue and blocking insulin receptors [300].

Overexpression of Gal-2 and Gal-4 in the placentas of women with GDM is also associated with changes in glucose-insulin regulation promoting insulin resistance, while Gal-12 acts as a negative regulator of lipolysis and its impaired function/deficiency in GDM may lead to increased fatty acid release, which deepens systemic insulin resistance [38,41,177].

Insulin resistance (IR) in gestational diabetes mellitus (GDM) is closely linked to placental and systemic mitochondrial dysfunction, which drives increased reactive oxygen species (ROS) production, inflammation, and reduced oxidative capacity [246,301]. Dysfunctional mitochondria in GDM trigger cellular stress and impair insulin signaling, creating a vicious cycle of metabolic disturbance. The above, contributing to trophoblastic cell dysfunction and increased inflammation through activation of the NOD-, LRR- and pyrin domain-containing protein 3 (NLRP3) inflammasomes. Higher concentrations of circulating cell-free mitochondrial DNA (cf-mtDNA) are associated with increased insulin resistance and can predict GDM diagnosis [301].

Galectins serve as critical regulators of mitochondrial homeostasis and can act in two directions, depending on the specific galectin and the cellular context [7,302]. For example, Gal-3, by maintaining communication between mitochondria and the ER, helps maintain the integrity of the mitochondrial network, among others by preventing apoptosis [303]. Gal-3 also appears to be essential for mitophagy, the process of cleansing cells from fragments of damaged mitochondria. Gal-3 relocalizes from the cytosol to “capture” damaged mitochondrial structures, ensuring they are properly recycled [304]. However, in severe COVID-19, epithelial Gal-3 has been found to inhibit mitochondrial respiratory chain genes in CD8+ T cells, leading to energy failure and thus limiting immune expansion [305].

Among the remaining galectins expressed in human placenta, Gal-1, Gal-7 and Gal-9 exhibit proapoptotic activity through interaction with Bcl-2 protein at the mitochondrial membrane, induction of mitochondrial membrane potential collapse, and/or the release of ROS, respectively [306,307,308]. The effect of Gal-8 on mitochondria, leading to their fragmentation, is associated with metabolic adaptation, in which a proliferative state is promoted by shifting the cell toward aerobic glycolysis (the Warburg effect), even while keeping the mitochondria functionally competent. Consistently, functional analysis under Gal-8 stimulation shows that “reprogrammed” mitochondria maintain an active electron transport chain, partially uncoupled from ATP synthesis, and an increased membrane potential, indicative of healthy mitochondria [309].

The influence of individual galectins on mitochondrial homeostasis in trophoblast cells and placental tissue in subsequent weeks of pregnancy complicated by the development of insulin resistance and GDM undoubtedly requires further research.

## 5. Concluding Remarks

Encoded in humans by 12 different genes, galectins are soluble carbohydrate-binding proteins capable of recognizing and binding to β-galactosides both intracellularly, at the plasma membrane level, and extracellularly. Galectins are described as molecular matchmakers because they orchestrate “functional pairing” between specific surface glycans and their receptors. As multivalent proteins, they do not just bind to individual sugar groups; they act as bridges that link multiple molecules together to form organized structures.

Most galectins are abundantly expressed within the maternal–fetal interface and placenta, with the dynamics of changes in the localization and expression patterns of individual galectins being developmentally regulated, demonstrating a relationship with trophoblast differentiation. Significant qualitative and quantitative changes in galectin distribution have been demonstrated in the placentas of pregnancies complicated by GDM. These changes can cause significant dysfunction of the diabetic placenta, which can be associated with many pathological processes, including chronic low-grade inflammation, impaired maternal–fetal immune tolerance, dysfunctional angiogenesis, and altered vascular remodeling during placentation, resulting in malperfusion due to insufficient trophoblast differentiation, migration and invasion into maternal uterine arteries. Ultimately, especially when poorly controlled glycemia is present, this translates into greater risks of poorer pregnancy outcomes for both the mother and neonate, including PE, FGR, cesarean section delivery, macrosomia, preterm birth, and neonatal hypoglycemia; GDM also increases the risk for postpartum type 2 diabetes and future cardiovascular disease in the mother.

However, additional studies are needed to clarify whether disturbances in placental galectin expression are secondary to altered metabolism in GDM or whether they are a primary cause of the pathomechanism of GDM-complicated pregnancy. The question of whether galectins collected from blood and simultaneously present in placental tissue (e.g., Gal-1, Gal-3, and Gal-9) can serve as prognostic biomarkers during pregnancy complicated by GDM also needs validation.

## Figures and Tables

**Table 1:** General characteristics and tissue distribution of members of the galectin family in humans, including in the maternal–fetal interface (marked in bold). aa—amino acids; dNKs—decidual natural killer cells; *LGALS*—lectin gene, galactoside-binding, soluble; MW—molecular weight; Treg—T regulatory cells; * given MW corresponds to the core protein, but glycosylation or oligomerization (dimerization, pentamerization) changes the observed mass in nature.

Galectin	Gene Name	Chromosomal Location of the Gene (Human)	Total aa Number in a Protein Chain	MW * (kDa)	Tissue Expression	References
Gal-1	*LGALS1*	22q13.1	135	14.72 (homodimer approx. 30)	Low tissue specificity: expressed in many tissues and cell types, including **decidua, endometrium, extravillous trophoblast (EVT) immune cells (i.e., dNKs and CD4^+^CD25^+^ Treg cells) and syncytiotrophoblasts (STBs)**	Wdowiak et al., 2018 [48]; Camby et al., 2006 [49]; Corral et al., 2022 [50]; Valero-Martínez et al., 2025 [51]; **von Wolff et al., 2005 [52]; Garín et al., 2007 [53]; Barrientos et al., 2014 [54]; Koopman et al., 2003 [55]**
Gal-2	*LGALS2*	22q13.1	132	14	gall bladder, gastrointestinal tract, exocrine pancreas, renal tubules, **placenta, decidua, endometrium, EVTs, STBs**	Wdowiak et al., 2018 [48]; Negedu et al., 2022 [56]; Thomsen et al., 2009 [57]; **von Wolff et al., 2005 [52]; Tian et al., 2015 [58]**
Gal-3	*LGALS3*	14q22.3	249	29–35 (pentamer approx. 150)	Low tissue specificity: expressed in many tissues and cell types, especially in immune and epithelial cells, and also in **cytotrophoblast (CTBs), endometrium and villous trophoblasts (VTs)**	Wdowiak et al., 2018 [48]; Yuan et al., 2025 [59]; **von Wolff et al., 2005 [52]; Bojić-Trbojević et al., 2019 [23]; Iglesias et al., 1998 [60]; Xiao et al., 2019 [61]**
Gal-4	*LGALS4*	19q13.2	323	37	gastrointestinal tract, gall bladder, **endometrium**	Johannes et al., 2018 [62]; Kozak & Zajkowska, 2025 [63]; **von Wolff et al., 2005 [52]**
Gal-7	*LGALS7*	19q13.2	136	15	squamous epithelia, skin, fetal heart, gastrointestinal tract, **decidua, endometrium, EVTs, glandular epithelial cells, STBs**	Johannes et al., 2018 [62]; Shimada et al., 2020 [64]; Teng et al., 2025 [65]; **von Wolff et al., 2005 [52]; Menkhorst et al., 2014 [66]**
Gal-8	*LGALS8*	1q43	317	35	Low tissue specificity: brain, liver, kidney, heart, lung, spleen, bone tissue (osteoclasts) and also in **endometrium, STBs, EVTs, VTs**	Johannes et al., 2018 [62]; Roy et al., 2024 [67]; Russo et al., 2025 [68]; **von Wolff et al., 2005 [52]; Kolundžić et al., 2011 [69]; Legner et al., 2024 [24]**
Gal-9	*LGALS9*	17q11.2	320	36	skin and gastrointestinal epithelial cells, liver, thymus, **endometrium, decidua, CTBs**	Brinchmann et al., 2018 [7]; Shimada et al., 2020 [64]; Valero-Martínez et al., 2025 [51]; **von Wolff et al., 2005 [52]; Popovici et al., 2005 [70]**
Gal-10	*LGALS10*	19q13.2	142	16.5	some fractions of hematopoietic cells and in subsets of leukocytes (eosinophiles) in several tissues, including **immune cells (i.e., CD4^+^CD25^+^ Treg cells) of** digestive tract, respiratory tract and **placenta**	Johannes et al., 2018 [62]; Li & Liu, 2025 [71]; **Buschmann et al., 2023 [72]**
Gal-12	*LGALS12*	11q12.3	336	38.4	adipose tissue, breast, **endometrium, STBs, EVTs**	Johannes et al., 2018 [62]; Tsao et al., 2025 [73]; **Buschmann et al., 2024 [41];** **von Wolff et al., 2005 [52]**
Gal-13	*LGALS13*	19q13.2	130	16	**exclusive placental expression, predominantly in STBs**	**Johannes et al., 2018 [62]; Balogh et al., 2019 [20]; Jovanović Krivokuća et al., 2021 [19]; Pei et al., 2023 [74]; Kliman et al., 2012 [75]; Than et al., 2004 [76]**
Gal-14	*LGALS14*	19q13.2	130–160	16–18	**predominantly expressed in placenta (STBs)**, eosinophiles	**Jovanović Krivokuća et al., 2021 [19]; Si et al., 2021 [77]; Balogh et al., 2019 [20]; Pei et al., 2023 [74];** Young et al., 2009 [78]
Gal-16	*LGALS16*	19q13.2	142	16.6	**exclusive placental expression**	**Than et al., 2009 [79]; Jovanović Krivokuća et al., 2021 [19]; Kaminker & Timoshenko, 2021 [80]; Pei et al., 2023 [74]**

**Table 2:** Changes in placental galectin expression in gestational diabetes mellitus (GDM) with regard to potential consequences for placental development and function.

Galectin	Expression Profile in GDM Placenta (vs. Normal Placenta)	Possible Consequences	References
Gal-1	Increased expression as a manifestation of an ineffective compensatory mechanism	Impaired placentation/placental development due to proinflammatory environment (chronic inflammatory background) resulting from disturbed maternal–fetal immune tolerance, altered trophoblast migration and invasion with poor spiral artery remodeling, increased risk of preeclampsia and FGR	Oravecz et al., 2025 [22]; Chen et al., 2022 [26]; Blois et al., 2014 [36]; Lapolla & Traldi, 2022 [282]; Roverso et al., 2016 [283]; Blois et al., 2019 [45]
Gal-2	Increased in CTBs and maternal decidua	Altered immunomodulatory effects (immune cell dysregulation), chronic low-grade inflammation, disturbed maternal–fetal immune tolerance, changed trophoblast migration and invasion, abnormal (compromised) placental angiogenesis, cardiovascular issues in the child	Chen et al., 2022 [26]; Hepp et al., 2020 [177]
Gal-3	Increased in EVTs	Impaired EVT functions, including inadequate trophoblast invasion and tube-like structure formation, that determine defective further placental development, FGR, increased inflammation in placental tissue, increased risk of cardiovascular disease in the mother in the future	Chen et al., 2022 [26]; Heusler et al., 2021 [37]; Negedu et al., 2022 [56]; Freitag et al., 2020 [285]; Briana & Malamitsi-Puchner, 2020 [286]; Zhang et al., 2021 [288]
Gal-4	Increased nuclear and cytoplasmic levels in decidua and STBs	Impaired trophoblast differentiation, maturation and adhesion influencing placentation, suboptimal control of cell migration, chronic local inflammatory response	Chen et al., 2022 [26]; Schrader et al., 2022 [38]; von Wolff et al., 2005 [52]; Arikawa et al., 2012 [201]
Gal-7	Upregulated expression in the nuclei and cytoplasm of decidual and STB cells	Local inflammation, impaired placentation due to abnormal trophoblast outgrowth resulting from changed trophoblast–endometrial epithelial cell adhesion and reduced placental production of angiogenic promoters	Chen et al., 2022 [26]; von Wolff et al., 2005 [52]; Menkhorst et al., 2020 [202]; Menkhorst et al., 2022 [203]; Seifert et al., 2024 [39]; Sewgobind et al., 2021 [291]
Gal-8	No data on placental expression in GDM	In case of altered expression: impaired adhesion, migration and invasion of primary CTBs, disturbed maternal–fetal immune tolerance	Legner et al., 2024 [24]; Kolundžić et al., 2011 [69]; Norambuena et al. [204]
Gal-9	No data on placental expression in GDM; in rodents, upregulated Gal-9 mRNA and protein expression in the labyrinth layer (the structure homologous to human chorionic villi) of GDM-affected placentas	In case of altered expression during the “implantation window”: impaired endometrial receptivity and disturbed maternal–fetal immune tolerance	Albuayjan et al., 2025 [40]; von Wolff M et al., 2005 [52]; Popovici et al., 2005 [70]
Gal-10	Increased, particularly in the decidual tissue, including immune decidual cells	Increased inflammatory response in placental tissue at the level of decidua	Jovanović Krivokuća et al., 2021 [19]; Blois et al., 2019 [45]; Buschmann et al., 2023 [72]
Gal-12	Overexpression in the nuclei of SCTs and EVTs	Increased local inflammatory response in placental tissue	Chen et al., 2022 [26]; Bushmann et al., 2024 [41]; Barbosa et al., 2025 [293]
Gal-13	Decreased expression in STBs and EVTs	Placental dysfunction due to suboptimal blood flow and delivery of oxygen and nutrients to the fetus due to defective remodeling of the spiral arterial vessels; local inflammation	Jovanović Krivokuća et al., 2021 [19]; Blois et al., 2019 [45]; Unverdorben et al., 2015 [34]; Vasilache et al., 2022 [206]
Gal-14	No data on placental expression in GDM	In case of altered expression: abnormal placental development due to both, altered trophoblast invasiveness and maternal–fetal immune balance with chronic low-grade inflammation	Balogh et al., 2019 [20]; Oravecz et al., 2025 [22]; Si et al., 2021 [77]; Wang et al., 2021 [296]
Gal-16	No data on placental expression in GDM	In case of altered expression: disrupted NF-κB-dependent signaling pathways influencing immune response, inflammation, apoptosis, cell growth and differentiation	Jovanović Krivokuća et al., 2021 [19]; Pei et al., 2023 [74]; Kamin-ker & Timoshenko, 2021 [80]; Kaminker et al., 2024 [297]

## Data Availability

No new data were created or analyzed in this study. Data sharing is not applicable to this article.

## Abbreviations

2′FL	2′-fucosyllactose
aa	amino acids
ADAM12	a disintegrin and metalloprotease 12
AGEs	advanced glycosylation end products
ALCAM	activated leukocyte cell adhesion molecule, also known as CD166
AMPK	5′ AMP-activated protein kinase
Ang2	angiopoietin-2
BCRs	B-cell receptors
BM	basal membrane
BMs	biomarkers
CBS	carbohydrate binding site
C-CRD	C-terminal carbohydrate recognition domain
CK7	cytokeratin 7
CLCs	Charcot–Leyden crystals
CLICs	clathrin-independent carriers
CRD	carbohydrate recognition domain
CTB	cytotrophoblast
CX3CL1	chemokine fractalkine
CX3CR1	chemokine CX3CL1 (fractalkine) receptor
DAMPs	pathogen-associated molecular patterns
dNKs	decidual natural killer cells
ECs	endothelial cells
EETosis	eosinophil extracellular trap cell death
EGFP	enhanced green fluorescent protein
EGPA	eosinophilic granulomatosis with polyangiitis
EL	endothelial lipase
enEVT	endovascular extravillous trophoblast
eNOS	endothelial nitric oxide synthase
EoE	eosinophilic esophagitis
ER	endoplasmic reticulum
ERK	extracellular signal-regulated kinase
ESCRT	endosomal complex required for transport
ET-1	endothelin-1
EVs	extracellular vesicles
EVT	extravillous trophoblast
FATP6	long-chain fatty acid transport protein 6, also known as solute carrier family 27 member 6 (SLC27A6)
FBV	fetal blood vessel
FGR	fetal growth restriction
FMc	fetal microchimerism (increased number of fetal cells in the mother’s blood)
FOXO1	forkhead box protein O1
Gal-1, Gal-2, Gal-3	galectins -1,-2,-3 and others, respectively
Galβ1-3GlcNAc	N-acetyllactosamine (LacNac) type 1
Galβ1-4GlcNAc	N-acetyllactosamine (LacNac) type 2
GBPs	glycan binding proteins
GDM	gestational diabetes mellitus
GDP	guanosine diphosphate
GlcNAc	N-acetylglucosamine
GLUTs	glucose transporters, also known as solute carrier 2A (SLC2A)
GTP	guanosine triphosphate
HOMA-IR	homeostatic model assessment—insulin resistance
hPL	human placental lactogen
HUVECs	human umbilical vein endothelial cells
IEC	intestinal epithelial cell
IFNγ	interferon gamma
IGFs	insulin-like growth factors
IGT	impaired glucose tolerance
IL-1β	interleukin-1 beta
ILV	intraluminal vesicle
IR	insulin resistance
IRS	immunoreactivity score
JAG1	Jagged-1, a critical Notch ligand in ECs
JNKs	Janus kinases
LacNac	N-acetyllactosamine
LAG3	lymphocyte-activated gene-3 (protein)
LDLs	low-density lipoproteins
*LGALS*	lectin, galactoside-binding or galectin encoding gene
LM	light microscopy
LPL	lipoprotein lipase
LPS	lipopolysaccharide
MAPKs	mitogen-activated protein kinases
MMPs	matrix metalloproteinases
mTOR	mechanistic target of rapamycin
MVBs	multivesicular bodies
MVEs	multivesicular endosomes
MW	molecular weight
N-CRD	N-terminal carbohydrate recognition domain
NDOs	non-digestible oligosaccharides
NDP52	nuclear dot protein 52 kDa, an autophagy receptor
NFAT	nuclear factor of activated T-cells
NF-κB	nuclear factor kappa-light-chain-enhancer of activated B cells
Notch	neurogenic locus notch homolog protein
OCLN	occludin
p38	p38 mitogen-activated protein kinase
PD-1	programmed cell death protein 1
PDPN	podoplanin
PGE_2_	prostaglandin E_2_
PKB	protein kinase B, also known as Akt
PMSCs	placenta mesenchymal stromal cells
PP13	placental protein 13, also known as galectin (Gal-13)
PPARs	peroxisome proliferator-activated receptors
PRRs	pattern recognition receptors
Rab11	small GTPase Ras-related protein Rab11
RAMPs	resolution-associated molecular patterns
RAS	renin-angiotensin system
RMSD	root mean square deviation
ROS	reactive oxygen species
Siglecs	sialic acid-binding lectins
sequestosome 1	autophagy receptor, also known as p62
sEVs	small extracellular vesicles
sFlt-1	soluble fms-like tyrosine kinase-1 or sVEGFR1
SLC27A6	solute carrier family 27 member 6, also known as long-chain fatty acid transport protein 6 (FATP6)
SLC2A	solute carrier 2A, also known as glucose transporters (GLUTs)
STBs	syncytiotrophoblasts
TCRs	T-cell receptors
TGN	trans-Golgi network
Th1	T helper 1 lymphocytes
TEM	transmission electron microscopy
TIM-3	T cell immunoglobulin and mucin domain-containing-3
TLR4	toll-like receptor 4
TLRs	toll-like receptors
TNFα	tumor necrosis factor alpha
Treg	T regulatory cells
TREM2	triggering receptor expressed on myeloid cells 2
TRIM16	tripartite motif-containing protein 16, an autophagy receptor
TSG101	tumor susceptibility gene 101, a component of the ESCRT-I complex
UPR	unfolded protein response
UPS	unconventional protein secretion or non-classical secretion pathways
VCTB	villous cytotrophoblast
VEGF	vascular endothelial growth factor
VEGFR2	vascular endothelial growth factor receptor 2
VSM	vasculosyncytial membrane
VSMC	vascular smooth muscle cell
